# Two- and Three-Dimensional In Vitro Models of Parkinson’s and Alzheimer’s Diseases: State-of-the-Art and Applications

**DOI:** 10.3390/ijms26020620

**Published:** 2025-01-13

**Authors:** Cristina Solana-Manrique, Ana María Sánchez-Pérez, Nuria Paricio, Silvia Muñoz-Descalzo

**Affiliations:** 1 Departamento de Genética, Facultad de Ciencias Biológicas, Universidad de Valencia, Calle Doctor Moliner 50, 46100 Burjassot, Spain; cristina.solana@uv.es; 2 Instituto Universitario de Biotecnología y Biomedicina (BIOTECMED), Universidad de Valencia, Calle Doctor Moliner 50, 46100 Burjassot, Spain; 3 Departamento de Fisioterapia, Facultad de Ciencias de la Salud, Universidad Europea de Valencia, Paseo de la Alameda 7, 46010 Valencia, Spain; 4 Instituto de Materiales Avanzados (INAM), Universidad de Jaume I, Avda Sos Banyat s/n, 12071 Castellón de la Plana, Spain; sanchean@uji.es; 5 Instituto Universitario de Investigaciones Biomédicas y Sanitarias (IUIBS), Universidad Las Palmas de Gran Canaria (ULPGC), Paseo Blas Cabrera Felipe “Físico” 17, 35016 Las Palmas de Gran Canaria, Spain

**Keywords:** Parkinson’s disease, Alzheimer’s disease, in vitro models, immortalised cell lines, iPSCs, organoids, engineering-based 3D models

## Abstract

In vitro models play a pivotal role in advancing our understanding of neurodegenerative diseases (NDs) such as Parkinson’s and Alzheimer’s disease (PD and AD). Traditionally, 2D cell cultures have been instrumental in elucidating the cellular mechanisms underlying these diseases. Cultured cells derived from patients or animal models provide valuable insights into the pathological processes at the cellular level. However, they often lack the native tissue environment complexity, limiting their ability to fully recapitulate their features. In contrast, 3D models offer a more physiologically relevant platform by mimicking the 3D brain tissue architecture. These models can incorporate multiple cell types, including neurons, astrocytes, and microglia, creating a microenvironment that closely resembles the brain’s complexity. Bioengineering approaches allow researchers to better replicate cell–cell interactions, neuronal connectivity, and disease-related phenotypes. Both 2D and 3D models have their advantages and limitations. While 2D cultures provide simplicity and scalability for high-throughput screening and basic processes, 3D models offer enhanced physiological relevance and better replicate disease phenotypes. Integrating findings from both model systems can provide a better understanding of NDs, ultimately aiding in the development of novel therapeutic strategies. Here, we review existing 2D and 3D in vitro models for the study of PD and AD.

## 1. Introduction

Neurodegenerative diseases (NDs) are a heterogeneous group of debilitating and incurable disorders characterised by the progressive degeneration of neurons, which results in central and peripheral nervous system damage and functional decline (reviewed in [1,2]). They are often influenced by genetic and ageing factors. Thus, lifespan extension worldwide is accompanied by an increase in the prevalence of NDs, representing an outstanding global public health issue (reviewed in [3]). Despite intensive research on NDs, the pathophysiological mechanisms underlying most of them are complex and poorly understood. Therefore, it is crucial to develop efficient research strategies to identify the pathological mechanisms underlying NDs, biomarkers for early diagnosis, and new therapeutic interventions to halt/slow disease progression (reviewed in [4,5]). The use of in vivo and in vitro models of NDs is pivotal to achieving these goals (reviewed in [6]). In this review, we focus on the most common NDs: Parkinson’s disease (PD) and Alzheimer’s disease (AD).

**Figure 1:**
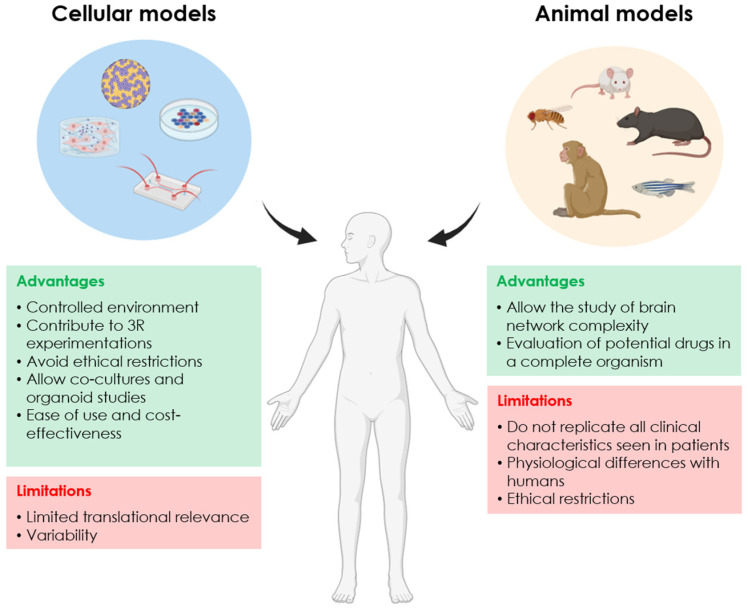
Main advantages and limitations of animal and cellular models for neurodegenerative diseases (NDs).

Animal models are essential in ND research since they allow the study of the brain network complexity involved in these disorders. Furthermore, the impact of potential therapies in drug development can be evaluated, testing the effectiveness, safety, selectivity, and availability of promising leads in a complete organism ([7], and reviewed in [8]). Genetic or neurotoxin-induced animal models of PD and AD have been developed in a wide range of species including rodents, non-human primates, and non-mammalian organisms such as *C. elegans*, *D. melanogaster*, and *D. rerio*. However, animal models have limitations as they do not replicate all of the clinical characteristics seen in patients. This is due to physiological differences with humans, which can cause clinical trial failure (reviewed in [8,9,10]). Therefore, the results must be interpreted with caution depending on the selected model. Moreover, some of these trials are expensive and ethically questionable (Figure 1).

In contrast, cellular models allow scientists to explore the characteristics of human diseases in a controlled environment. They contribute to replacing, reducing and refining animal experimentation, thus avoiding the ethical restrictions and costs associated with animal models (reviewed in [11,12]). Moreover, in vitro models are also effective in drug development. In such models, the identification of cell types involved in the disease of interest and how they interact is essential to mimic healthy/diseased tissue or organs (reviewed in [11,12]) (Figure 1). Considering the cell type heterogeneity involved in PD and AD, distinct types of 2D and 3D in vitro models of both diseases will be discussed below.

## 2. Parkinson’s and Alzheimer’s Disease: The Most Prevalent Neurodegenerative Diseases

PD is the most prevalent motor disorder globally. The pathological hallmark of PD is the progressive degeneration of dopaminergic (DA) neurons in the *substantia nigra* of the brain, sometimes accompanied by misfolded α-synuclein (α-syn) accumulation in Lewy bodies. The neuropathology involves multiple other motor and non-motor circuits that affect patients’ life quality (reviewed in [13]). Several studies have also demonstrated that dysfunctional glial cells, such as astrocytes [14] and microglia [15], play a crucial role during PD pathogenesis and are responsible for inflammation-driven neurodegeneration (reviewed [16]). Typical motor symptoms associated with the disease include resting tremor, rigidity, postural instability, bradykinesia, and walking difficulties. Non-motor symptoms are also evident in PD patients such as neuropsychiatric and cognitive alterations, dysphagia, constipation, and sleep disturbances, which often precede the motor symptoms by years or even decades (reviewed in [17,18]). The exact molecular mechanisms underlying neuronal loss have not been fully elucidated. However, several factors impact PD onset and progression such as impaired protein clearance, mitochondrial dysfunction, oxidative stress, metabolic alterations, calcium dyshomeostasis, and neuroinflammation. Different pathways involved in PD pathogenesis have been identified by analysing the function of genes involved in rare familial forms of the disease (reviewed in [19]), which constitute around 10–15% of PD cases. Among them, there are three genes that are well validated to cause autosomal dominant PD forms like *Synuclein Alpha* (*SNCA*), *leucine rich repeat kinase 2* (*LRRK2*)*,* and *vacuolar sorting protein 35* (*VPS35)*, and three causing autosomal recessive forms: *Parkin* (*PRKN*), *PTEN-induced kinase 1* (*PINK1*), and *DJ-1* (also known as *PARK7*). Other genes have been reported in small numbers of cases or families (reviewed in [20]). However, most PD cases are sporadic, and the aetiology is multifactorial with both genetic and environmental factors playing a role. In fact, the incidence of PD is greater in individuals exposed to pesticides and traumatic brain injury (reviewed in [18,20]).

AD, the most common ND, is characterised by the formation of extracellular amyloid-β (Aβ) plaques around neurons, and intracellular neurofibrillary tangles (NFT) of hyperphosphorylated Tau (P-Tau), affecting neuronal function and resulting in their progressive death. Aβ plaque formation disrupts the endothelial cells forming the blood–brain barrier, in addition to surrounding astrocytes, rendering it highly permeable (reviewed in [21,22]). AD was proposed to be caused by a loss of cholinergic (Ach) neurons in the septal area (reviewed in [23]). However, several types of neurons in the hippocampus, microglia, astroglia, oligodendrocytes, and altered vasculature appear to contribute to disease onset and progression [24]. Also, neuroinflammation and insulin resistance are strongly associated with AD onset and progression. In the brain, the role of insulin is key for maintaining optimal cerebral blood flow, inflammatory responses, oxidative stress levels, Aβ clearance, Tau phosphorylation, apoptosis, lipid metabolism, transmitter–receptor trafficking, synaptic plasticity, and memory formation (reviewed in [25,26]). In fact, among other kinases, glycogen synthase kinase-3β (GSK-3β) is fundamental in Tau phosphorylation. GSK-3β is disinhibited upon insulin resistance, thus resulting in P-Tau (reviewed in [27]). Mitochondrial dysfunction and altered mitophagy are also considered critical factors in AD pathogenesis (reviewed in [28]). AD patients typically exhibit behavioural and cognitive dysfunction with variable severity, leading to dementia (reviewed in [29]). Age and exposure to environmental toxins are the most important risk factors in the development of sporadic or late AD, in which the first symptoms appear in people over 70 years. In this AD form, genetic factors may contribute to increased susceptibility. Although most AD cases are sporadic, there is a less frequent form classified as familial AD, which is associated with mutations in specific genes, including those encoding amyloid-β precursor protein (APP), presenilin 1 (PSEN1), and presenilin 2 (PSEN2). This dominantly inherited AD form represents less than 5% of all AD cases and appears about 40 years earlier than sporadic AD, but both share many clinical biomarkers and pathological features [29].

## 3. Two-Dimensional In Vitro Models to Study Parkinson’s and Alzheimer’s Disease

**Figure 2:**
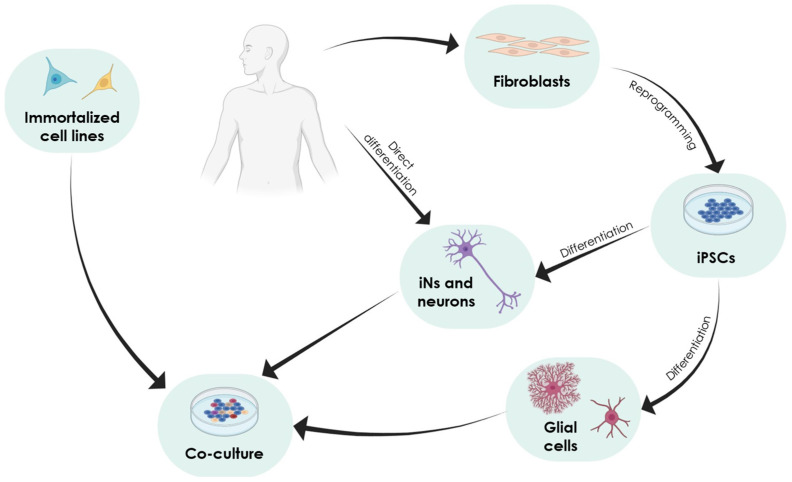
Scheme of the workflow for using 2D cell culture to study NDs. To establish co-cultures, fibroblasts from patients with NDs are isolated and reprogrammed into iPSCs. These cells can be differentiated in neurons and/or glial cells and used in co-cultures. Another strategy is to directly differentiate the somatic cells of ND patients into neurons (iNs) or to use immortalised cell lines in co-cultures.

In vitro approaches have been extensively used to understand the aetiology and pathogenesis of PD and AD. As mentioned above, PD-associated phenotypes are due to DA neuron death. However, it has been shown that glial cells exert a neuroprotective effect. Specifically, cell death can be alleviated by the inhibition of microglial cell activation [15], or by neurotrophic factors and antioxidative stress molecules secreted by astrocytes [30]. In this section, we will focus on 2D in vitro models that have been applied to study PD and AD. Among them, we mention classical immortalised cell lines [31] but also human induced pluripotent stem cells (iPSCs) [32], or neurons directly derived from somatic cells [33]. These cells can be cultured in 2D layers individually, but also mixed in co-cultures, in which various cell types are grown on a surface forming a monolayer (reviewed in [34]). Co-culturing different cell types has emerged as a promising strategy to investigate the complex mechanisms underlying ND pathophysiology, since they allow modelling interactions between neurons, glial cells, and other components of the microenvironment (Figure 2).

### 3.1. Immortalised Cell Lines

#### 3.1.1. SH-SY5Y Cells

The SH-SY5Y cell line, derived from human neuroblastoma, has become a fundamental tool for studying PD and AD. This line has undergone various selection rounds to improve its adaptability for research purposes (reviewed in [35]). SH-SY5Y cells can be differentiated into neuron-like cells using agents such as retinoic acid (RA).

SH-SY5Y cells are characterised by a catecholaminergic phenotype, meaning they can synthesise neurotransmitters like dopamine and noradrenaline, rendering them useful for modelling the DA neurodegeneration seen in PD (reviewed in [36]). Indeed, SH-SY5Y cells express key enzymes involved in dopamine synthesis, such as tyrosine hydroxylase (reviewed in [35]). The RA-mediated differentiation of SH-SY5Y cells into neuron-like cells and subsequent treatment with neurotoxins allows the molecular mechanisms underlying DA neurodegeneration to be studied [37]. For example, exposure to neurotoxins like MPP+, a metabolite of MPTP (1-methyl-4-phenyl-1,2,3,6-tetrahydroperydine) (reviewed in [35]), or rotenone mimics the oxidative stress effect and mitochondrial impairment seen in sporadic PD (reviewed in [35,36]).

The genetic manipulation of SH-SY5Y cells has led to the generation of models replicating key pathological features of familial forms of PD. For example, knocking down the *DJ-1* gene in SH-SY5Y cells provides insights into the role of oxidative stress and mitochondrial dysfunction in the disease [38]. Other familial PD models include silencing or overexpressing genes like *PINK1* and *LRRK2*. Silencing *PINK1* in SH-SY5Y cells results in mitochondrial dysfunction, while overexpressing mutant forms of *LRRK2* leads to α-syn aggregation, both critical for understanding familial PD (reviewed in [35]). Additionally, *α-syn* overexpression models have been used to study protein aggregation, a hallmark of both sporadic and familial PD forms (reviewed in [36]). These models allow researchers to investigate the cellular mechanisms underlying PD, providing insights into oxidative stress imbalance, mitochondrial dysfunction, and protein aggregation.

RA-differentiated SH-SY5Y cells are also widely used in AD research, specifically for Tau pathology (reviewed in [39]). Although SH-SY5Y-derived neurons lack clearly defined neuronal subtypes, several differentiation protocols enhance the expression of different neuronal markers. For instance, SH-SY5Y cells can be differentiated to an Ach neuronal phenotype after RA exposure and followed by brain-derived growth factor (BDNF) incubation [40]. Given the great versatility and accessibility of differentiation, SH-SY5Y cells provide a versatile platform for studying protective agents to restore the neuronal damage caused by Aβ toxicity ([41,42] and reviewed in [39]).

#### 3.1.2. PC12 Cells

The PC12 cell line, derived from a rat pheochromocytoma tumour, is also widely used for studying NDs. Originally established by Greene and Tischler in the 1970s, PC12 cells have the unique ability to differentiate into neuron-like cells upon stimulation with nerve growth factor (NGF), which enhances their suitability as a neuronal model [43,44]. Once differentiated, these cells acquire features of sympathetic neurons, including the development of neurite outgrowth, making them a valuable tool for investigating neuronal signalling, survival, and death [44,45].

In PD research, PC12 cells have been employed to create various models mimicking the DA neurodegeneration observed in this disease. For instance, exposure to neurotoxins such as 6-hydroxydopamine (6-OHDA) and MPP+ leads to mitochondrial dysfunction and oxidative stress, key features of sporadic PD pathology [46]. Additionally, other studies have utilised these cells to investigate familial forms of PD by manipulating *PINK1* and *PRKN*. By knocking down or overexpressing these genes in PC12 cells, researchers have been able to model mitochondrial impairments that mirror those seen in PD patients [47]. Another study performed in PC12 cells has shown that the *PINK1*/*Parkin* pathway antagonises with PGC-1α to regulate mitochondrial biogenesis, fission/fusion, and mitophagy, therefore contributing to the maintenance of mitochondrial homeostasis in rotenone-induced neurotoxicity [48]. Finally, the key role of *LRRK2* in controlling vesicle trafficking and distribution has also been investigated using PC12 cells [49].

PC12 cells have been applied to AD research due to their ability to model Aβ-induced toxicity. When exposed to Aβ oligomers, differentiated PC12 cells exhibit increased oxidative stress levels and neuronal death, providing a useful platform for studying the molecular mechanisms underlying Aβ toxicity and its contribution to AD progression (reviewed in [50]). This model has been instrumental for exploring potential therapeutic approaches aimed at reducing oxidative damage and preventing Aβ-induced neuronal loss [45].

#### 3.1.3. LUHMES Cells

The LUHMES (LUnd Human MESencephalic) cell line, derived from human foetal mesencephalon, has been used as a powerful tool for studying NDs. Immortalised through the insertion of the *v-myc* gene under a tetracycline-inducible system, LUHMES cells are easily differentiated into mature DA neurons upon exposure to tetracycline, cyclic AMP (cAMP), and glial-derived neurotrophic factor (GDNF) [51,52].

Once differentiated, LUHMES cells closely mimic primary DA neurons, displaying similar biochemical and morphological characteristics, including the expression of tyrosine hydroxylase and dopamine transporters [53]. In the context of PD, LUHMES cells have been used extensively to model the neurodegenerative effects caused by toxins like MPP+ [53,54,55]. These cells have also been utilised to study genetic forms of PD, particularly those related to mutations in the *LRRK2* gene [51]. LUHMES cells provide a robust platform for understanding LRRK2 biology and for screening potential therapeutic compounds aimed at mitigating PD-related neuronal degeneration.

Some studies used them for AD since they secrete Aβ [56]. They have also been used to investigate the role of the insulin signalling pathway in co-cultures with astrocytes [57].

### 3.2. iPSC-Derived Cells

iPSCs represent an interesting model to study a disease with the genetic background of the human patient. The generation of iPSCs begins with the acquisition of somatic cells from the patients, usually fibroblasts. After establishing the cultures, cells are reprogrammed using viral derived vectors (e.g., Sendai virus or lentivirus), that will carry the genes to express the transcription factors, also known as the *Yamanaka factors*, namely, *Oct3/4*, *Sox2*, c-*Myc*, and *Klf-4*, that are required to transform the fibroblast into iPSCs [32]. Moreover, wild-type iPSCs can be genetically modified to overexpress or inhibit genes of clinical interest. Finally, this technology allows enhanced iPSC-derived cells to be created, with improved clearance for specific toxic peptides, for instance, or even personalised medicine, correcting the patient iPSC lines back to a healthy gene variant. More research is still to be conducted to achieve this exciting application of iPSCs.

In addition to their application in studying the pathological mechanisms underlying NDs or the effect of specific gene mutations associated with patients and correlation to behaviour, autologous gene-edited iPSC-derived cells transplanted into patients may open new therapeutic prospects (reviewed in [58]). For PD, a seminal paper showed that human iPSC-derived DA neurons can survive in a primate (*Macaca fascicularis*) model of PD, induced by MPTP exposure [59]. Also in murine brains, iPSC-derived microglia cells were successfully integrated in an AD brain model. Interestingly, microglial cells derived from iPSC xenotransplants were able to migrate and extend processes towards Aβ plaques to phagocytose them, thus showing the potential of this strategy in clinical applications [46].

#### 3.2.1. iPSC-Derived Neurons

Several studies have been performed with DA neurons differentiated from iPSCs, derived from patients with familial forms of PD. As an example, DA neurons with mutations in *PINK1* and *PRKN* genes, both involved in mitochondrial homeostasis, exhibited abnormal mitochondrial function [60]. Remarkably, other studies using iPSC-derived DA neurons from patients with monogenic *LRRK2*-linked familial PD or sporadic PD, and healthy subjects have demonstrated clear epigenetic changes associated with the disease [61]. These results indicate that certain epigenetic alterations associated with the disease are only strongly linked to the DA neuronal type. Other methods of studying genes involved in PD have used neural stem cells (NSCs) differentiated from iPSCs (ipsNSCs). The correction of an *LRRK2* mutation in these differentiated neurons was shown to rescue the ageing process [62].

Traditionally, AD was not associated with a specific neuronal type. Hence, most protocols using iPSCs to model AD differentiated them to neuron-like cells to study specific mutations. Thus, an early study using fibroblasts from AD patients showed that iPSC technology can be used to observe AD-relevant phenotypes, such as pathological levels of the Aβ, P-Tau, and GSK-3β [63]. Interesting validations for this model have been obtained with human-derived iPSCs with familial-AD-associated mutations such in the *PSEN1* and *APP* genes. Mutant-derived neuronal cells exhibited abnormally increased electrical activity when compared with their isogenic healthy controls [64]. Several mutations associated with familial and sporadic AD have been validated with iPSCs differentiated into cortical neurons (reviewed in [65]).

Sporadic AD has also been studied using iPSCs to identify specific proteins and pathways involved in the disease. To this end, iPSC lines from 53 individuals (postmortem) including normal ageing and AD diagnosed were differentiated into cortical neuronal type cells. In these differentiated cells, RNA and protein profiles, as well as Aβ products and Tau species, were evaluated [66]. This study not only identified specific proteins and pathways associated with AD risk, neuropathological burden, and cognitive trajectory in the donors, but also validated these findings with the results of brain tissue analysis from the same donors. Perhaps the most striking association was phosphatase 1 (PP1) levels with late-onset AD risk, and the levels of APP cleavage products and P-Tau levels.

#### 3.2.2. iPSC-Derived Glial Cells

Glial cells, such as microglia and astrocytes, provide support for neurons and serve as the resident brain immune cells. While microglial cells originate from the mesoderm and are derived from primitive macrophages in the yolk sac, astrocytes have a neuroectodermal embryonic origin [67,68]. It is known that microglial cells are primarily involved in regulating neural inflammation, which plays a significant role in the start and progression of NDs. Further, astrocytes are neuron-supporting cells that harbour a powerful neuroprotective arsenal including neurotrophic factors and antioxidants. It has been shown that neurodegeneration can be alleviated either by the inhibition of microglial cell activation [15], or by the action of astrocyte-secreted factors [30]. However, glial cells can also alter the pathology associated with many NDs, including PD and AD (reviewed in [67]).

Different iPSC-based cell models have been generated to examine the role of microglia and astrocytes in familial PD. As an example, iPSC-derived *PRKN*-deficient glial cells were obtained from PD patients and healthy donors to study the response to TNF-α stimulation [68]. This study has shown that glial cultures from PD patients are less reactive compared to those from healthy donors, indicating a reduced activation capacity in *PRKN*-deficient glia [68]. Recently, iPSCs were also obtained from a patient carrying a pathogenic genetic variant the *GBA1* gene, which were efficiently differentiated into astrocytes [69]. It was demonstrated that this mutation caused a decrease in β-glucocerebrosidase enzyme activity, leading to lysosomal dysfunction. This cell model will allow the contribution of astrocytes to *GBA1*-associated PD pathogenesis to be studied in co-cultures with *GBA1* mutant DA neurons.

Regarding microglial cells’ relevance in AD research, there is compelling evidence of an association between AD and mutations of genes highly or exclusively expressed in such cells (reviewed in [70]). Thus, several methods have been reported to differentiate iPSCs into microglia lineage to study AD pathophysiology (reviewed in [71]). As an example, a specific protocol has been used to study and compare the molecular phenotypes and functional consequences of different Apolipoprotein E (ApoE) isoforms in iPSC-derived neurons, astrocytes, and microglia [72]. In addition, the triggering receptor expressed on myeloid cells 2 (TREM2) role in AD has been studied in detail in several reports using iPSC-derived microglia (for a review, see [73]). Evidence from genetic studies have identified rare genetic variants as strong risk factors for developing AD (reviewed in [74]).

### 3.3. Neurons Directly Derived from Somatic Cells

Neurons directly derived from somatic cells, known as induced neurons (iNs), have emerged as a new tool for modelling NDs [33]. Unlike iPSCs, which must first be reverted to a pluripotent state before differentiation, iNs are generated by directly reprogramming somatic cells, such as fibroblasts, into functional neurons. This approach offers several advantages, including faster and more efficient neuron generation while avoiding the intermediate pluripotent stage, which may induce tumorigenicity (reviewed in [75]). iNs display mature neuron electrophysiological properties, including the ability to form functional synapses and respond to neurotransmitters. These features render them a highly relevant model for studying neuronal function and dysfunction in NDs [76].

In PD research, iNs have been particularly valuable for studying patient-specific disease mechanisms. For example, fibroblasts from patients with genetic mutations linked to PD, such as *LRRK2* or *SNCA*, allowed researchers to investigate how these mutations contribute to DA neuronal death and mitochondrial dysfunction [75]. These patient-derived iNs also provided a platform for drug testing, screening therapeutic compounds to identify treatments that may prevent or reverse neurodegenerative changes (reviewed in [77]).

iNs have been used to model AD. Reprogramming fibroblasts from patients with familial AD mutations (such as those in *APP* or *PSEN1*) into iNs allows Aβ and Tau pathologies to be studied in a human context. These studies offers insights into the mechanisms driving neuronal death and synaptic dysfunction in AD. iNs derived from AD patients have also been used to identify compounds that reduce Aβ production or prevent Tau phosphorylation, both of which are central therapeutic targets in the disease [78].

### 3.4. Advantages and Disadvantages of 2D In Vitro Models

Immortalised cell lines such as SH-SY5Y, PC12, and LUHMES remain essential tools in ND research due to their ease of use and cost-effectiveness. They maintain good batch-to-batch consistency, are less expensive to maintain, and can be applied in high-throughput screening assays [51,53]. These cell lines are robust and can be differentiated into neuron-like cells or mature DA neurons (LUHMES). This makes them valuable for high-throughput drug screenings ([38] and reviewed in [36,79]), the study of disease mechanisms ([38,45,46,80,81], and reviewed in [35,36]), or to model familial PD [51,82]. However, immortalised cell lines have limitations. Due to their cancerous origins, they may not accurately replicate the genetic and phenotypic complexity of human neurons, which limits their translational relevance. This variability affects the differentiation efficiency of SH-SY5Y into DA neurons and response to toxins (reviewed in [36]). Another limitation for PC12 cells is their rat origin that limits the direct applicability of findings to human disease, as species-specific differences can influence the results.

On the other hand, iPSC-derived neurons or glial cells offer a more accurate representation of human neuronal physiology, especially when derived from patients with known genetic mutations associated with PD or AD. These models allow researchers to study the specific genetic underpinnings of neurodegeneration, providing a more relevant system for testing potential therapies. However, iPSC-based models come with their own challenges, including higher costs, technical complexity, and variability between cell lines derived from different patients [75].

Despite their advantages, iNs are not without limitations. One major challenge is that reprogramming efficiency can vary depending on the source of the somatic cells and the specific combination of transcription factors used. Additionally, while iNs offer a faster route to generate neurons than iPSCs, they may lack the long-term proliferative capacity of iPSC-derived neurons, hindering the production of large quantities of neurons for certain types of studies [75]. Moreover, the direct reprogramming process may bypass important developmental stages, potentially affecting the maturation and functional properties of the resulting neurons (reviewed in [77]). However, iNs offer the unique advantage of being able to model patient-specific disease mechanisms in a time-efficient manner, while avoiding the technical complexity and potential risks associated with iPSCs [75]. As techniques for generating and studying iNs continue to improve, these cells are likely to become an increasingly valuable tool for unravelling the cellular and molecular mechanisms underlying PD, AD, and other NDs.

In conclusion, while immortalised cell lines, iPSC-derived neurons, or iNs each have distinct advantages and limitations, their use in combination with animal models provides a comprehensive approach to studying NDs. By integrating these models with cutting-edge technologies such as co-cultures and 3D systems, researchers can gain deeper insights into the pathophysiology of diseases like PD and AD and ultimately improve therapeutic strategies.

## 4. Three-Dimensional In Vitro Models of Parkinson’s and Alzheimer’s Disease

**Figure 3:**
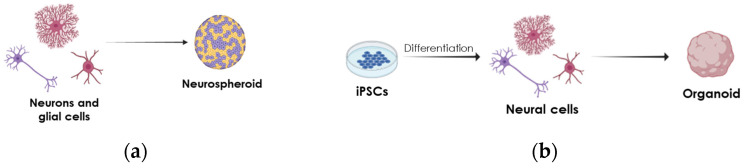
Schematic of biological-based strategies used to generate 3D cultures. (**a**) Neuronal cells can be cultured together to generate neurospheroids. (**b**) Method involving neurons and glial cells derived from patient’s iPSCs and extracellular matrix (Matrigel) to develop midbrain or brain organoids.

As mentioned above, in PD and AD, multiple cell types are affected. Hence, any in vitro biological relevant model should not only include DA or ACh neurons, but also the associated cells. While these heterogeneous cell cultures can be accomplished in 2D (reviewed in [83]), multiple studies have shown that 3D cell cultures are better to model diseases (reviewed in [84]). Therefore, sophisticated 3D culture models with high cell diversity have been developed to study such interactions in the context of NDs (reviewed in [6]). In this section, we will focus on reviewing 3D cell cultures used to model PD and AD, preferably including associated cell types. The early protocols to model PD or AD in vitro using 3D cultures adapted 2D differentiation protocols using different starting cell populations to form spheroids. Spheroids are compact aggregates of cells formed by mainly only one cell type. Neurospheroids mainly formed by DA neurons, with few astrocytes present, have been used to model PD (Figure 3a) [85,86]. Likewise, spheroids containing cortical neurons have been used to model AD [87]. While spheroid generation is robust, simple, cost-effective, and allows the generation of enough homogeneous samples for high-throughput screens, it lacks the cellular complexity needed to model all cellular PD and AD components.

**Figure 4:**
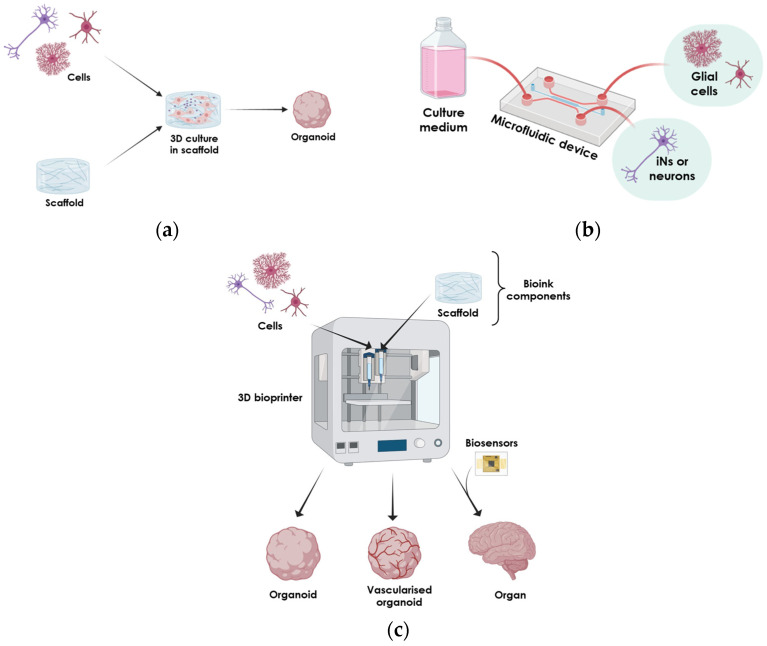
Schematic of engineering-based strategies used to generate 3D cultures. (**a**) Cells cultured in synthetic materials (scaffolds) to generate organoids. (**b**) Microfluidic devices to investigate PD or AD. (**c**) Bioinks can be used together with cells to form complex organoids that can even have a vascular system if endothelial cells are added. The use of biosensors to monitor the environment and feedback into organ activity can lead to better in vitro 3D models for NDs.

Below, we present 3D neural cell culture systems developed following a cellular-biology-based approach to generate organoids (Figure 3b), or from an engineering-based approach applying scaffold-based, microfluidic-based, and bioprinting systems (Figure 4, reviewed in [88]).

### 4.1. Organoid Models

Organoids are complex 3D structures in spatially defined patterns based on single cell suspensions to form aggregates using specific differentiation media and Matrigel (Figure 3b). Over 10 years ago, cerebral organoids from iPSCs or human embryonic stem cells (hESCs) were developed [89,90,91]. However, these early protocols only produced cortical neurons. Since those early days, multiple protocols and methods have been developed to improve brain organoid formation and to model other brain regions (see Figure 4).

#### 4.1.1. Midbrain Organoids for Parkinson’s Disease

Of special interest for PD are the midbrain organoids (MOs) as they include DA neurons [92,93,94,95]. While none of these studies tested for the presence of microglia, some showed that astrocytes are present. However, none used their MOs to study how the astrocytes are involved in PD. For a full timeline of key milestones in methods to derive MOs, see [96].

Recent protocols allow the formation of more relevant MOs to PD as they include DA neurons together with functional astrocytes [97]. The more advanced protocol (DAC3.0) produces neuromelanin-like granules resembling the *substantia nigra* of the brain, where DA neurons accumulate. The presence of astrocytes allowed the authors to model PD by treating the MOs with MPTP, metabolised to MPP+ in the astrocytes, causing DA neuron cell death (reviewed in [98]). The same group improved their protocol, simplifying it, to generate MOs amenable to high-throughput screens [99]. The new protocol generates uniform MOs containing mature DA neurons as indicated by their production of neuromelanin-like granules. The authors modelled PD using again MPTP and rotenone. Other studies have also been interested in analysing the presence of astrocytes in MOs [100,101,102,103,104]. Given the importance of astrocytes and microglia for PD, the latest protocols of interest include the presence of these cell types together with DA neurons [105,106,107,108].

The best 3D models capturing the multicellular involvement in PD progression, and that are easier to generate, come from cell biology approaches. BrainSpheres are composed by mature neurons and glial cells (astrocytes and oligodendrocytes) that reproduce neuron–glial interactions and connectivity [109]. Their main advantage is their ease of production and the regular size of the individual spheres, homogenising the results. Another approach includes the recent so-called assembloids (reviewed in [110]). Assembloids are usually generated by the co-culture and fusion of spheroids or organoids from the different brain regions or cells of interest. Multiple attempts have been made to include microglia in human brain organoids (reviewed in [111]) and even midbrain organoids [112]. A more advanced version of the latter study based on the air–liquid interphase culture of MO slices together with microglia allowed some long-term culture (over 180 d) [113]. In this system, even astrocytes appear after 180d in culture when microglial cells are no longer detected. Future studies using these methods in PD or AD contexts will improve disease modelling.

#### 4.1.2. Brain Organoids for Alzheimer’s Disease

As mentioned above, AD is not associated with specific cell population death, but more related to several cerebral areas. Therefore, researchers rather seek to model the brain architecture than differentiate cells into specific cell types. Organoids have been published with differentiated SH-SY5 that have been shown to resemble neurons like those in Matrigel scaffolds [80]. However, personalised organoids, containing glia and neurons, using patients’ induced pluripotent cells have been established to allow the study of both sporadic and familiar forms of AD (reviewed in [114,115]). Also, advances in organoid development with a functional vascular system upon in vivo transplantation have been achieved [116]. Some studies have shown that non-genetic stimulus (toxic environment) leads to a time-dependent accumulation in Aβ production and a higher Aβ42/40 ratio in organoids, suggesting that brain organoids can effectively model non-genetic causes of the disease [117].

The unique spherical structure of brain organoids promotes the accumulation of Aβ, allowing for the formation of plaques and the simultaneous development of NFT pathology. Hence, human brain organoids can recapitulate Aβ and Tau pathological features ([114] and reviewed in [118]). Furthermore, this system is particularly valuable for studying the neuroinflammatory aspects of AD, as it can be co-cultured with microglia and astrocytes, offering insights into the inflammatory responses involved in the disease. Moreover, since significant differences in transcriptomics between mice and humans are found, human-cell-derived organoids allow for transcriptomic analysis more relevant to human disease. Altogether, this provides further advantages over animal transgenic models of AD (reviewed in [119]).

Brain organoids with patient-derived cells have allowed connections to be established between abnormal APP metabolism and increased mitochondrial vulnerability to oxidation [120], thus providing tools for tailored treatments. Furthermore, the use of 3D approaches has allowed the progression to 4D analysis. A recent study has shown a key regulating mechanism in the pathophysiology of AD using 4D analysis. In this report, a TREM2-dependent phagocytosis has been shown to be primed by externalised Phosphatidylserine (ePtdSer) in the vicinity of Aβ plaques [121]. ePtdser was previously well known for its role during development as an “eat me” signal to macroglia in the pruning process. Interestingly, damaged neurons also expose ePtdSer. In this study, 3D organoids were analysed over time (4D) to elucidate the biological processes involving TREM2-dependent Aβ phagocytosis [121]. Hence, the use of these cerebral organoids, together with image analysis software, represent a significant step forward in the study of the complex mechanisms underlying AD and other NDs (reviewed in [122]).

#### 4.1.3. Advantages and Disadvantages of Organoid Models

The main advantage of organoids is that they better replicate the in vivo architecture of the human brain (or midbrain), including the differentiation of cortical layers, which 2D cultures cannot achieve. This 3D setup enables complex, multidirectional cellular interactions, providing a more accurate representation of PD and AD. As indicated above, they better mimic the diseases’ cellular and molecular aspects than 2D models. Due to all of these advantages, 3D is a valuable platform to model PD and AD that is rapidly expanding and increasingly being reviewed [79,123,124,125,126].

The main caveat of these approaches is that they can be cultured only for a short period. When attempting to culture them long term, necrotic cores appear [127]. To overcome this, two approaches have been followed: the inclusion of a blood supply or the use of spinning bioreactors. To date, several attempts have been made to include endothelial-like or some type of vascularised structures in brain organoids [128,129,130] or forebrain assembloids [131]. However, none show circulating blood for nutrient supply that would allow long-term culture and avoid cell death, unless they are transplanted into an animal model. Furthermore, this type of assembloid or co-culture with endothelial cells has not been achieved in MOs. The main aim of spinning bioreactors is to enhance the diffusion of oxygen and nutrients without adding vasculature. This method allows the generation of bigger cortical structures and a longer incubation time (up to a year), preventing cell death in the organoid core [132,133].

Another issue with organoid generation is the availability of multiple published protocols with few details, which affects their reproducibility or efficiency. Hence, it can often be difficult to replicate them in different labs or even compare the results obtained in different labs. Furthermore, the use of Matrigel, whose composition varies from batch to batch, adds extra variability to the process. As with the 2D model, another disadvantage when using patient-derived cells to generate cerebral organoids is the high variability from cell to cell. This caveat has been highlighted using reprogrammed human fibroblasts from individuals with different APOE-ε genotypes to generate isogenic lines with different APOE profiles, mainly due to the different differentiation process among cell batches [134].

Finally, in all forms of dementia, the major and more important risk factor is age. iPSCs used to generate the organoids are thought to be at a foetal stage (reviewed in [135,136]). Thereby, the big question that arises is that, if they are aged in the laboratory, can they be considered to be equivalent to the effect of human ageing? In other words, one of the major disadvantages of organoids is their limited ability to recapitulate the mechanisms by which ageing contributes to dementia.

### 4.2. Engineering-Based 3D Models

Advances in bioengineering have also led to more sophisticated models using co-cultures in 3D, and these will be discussed here. These models are based on culturing (or co-culturing) cells of interest in 3D using non-commercial biocompatible materials created ad hoc or using devices (Figure 4). The main goal is to maintain the 3D structure and/or to better control the microenvironment and nutrient access (reviewed in [137]). The cultured cells establish more physiologically relevant connections, hence being better able to model certain disease aspects.

This approach also pursues the use of biocompatible materials, which would allow these scaffolds to be used with functional cells that would engraft in the receiving brain and, hence, be used for cell replacement therapy (Figure 4a. This has been tested when observing cell outgrowth and survival in mouse ex vivo and in vivo models [138]. In PD research, biocompatible 3D scaffolds have been employed using PC12 cells to support neuronal growth, mimicking more physiologically relevant environments [43]. Additionally, the use of 3D co-cultures with LUHMES cells has proven valuable for long-term neurotoxicity and cellular resilience study in PD models [139]. iPSC culture and differentiation towards DA neurons in a 3D structure better represent the molecular mechanisms in PD [140]. The scaffold composition used in PD models include silk hydrogel [140]; PureMatrix [141]; poly(desaminotyrosyl tyrosine ethyl ester carbonate) (pDTEc) electrospun fibres [138]; and carbon fibres [142]. In AD research, 3D models often focus on creating a simplified disease representation to study Aβ aggregation, Tau phosphorylation, or drug screening in a more biologically relevant context. Materials like microfiber scaffolds have been shown to accelerate neuronal differentiation compared to 2D cultures [143]. In this study, parameters like scaffold porosity and hydrophobicity were crucial for ensuring an optimal microenvironment. Interestingly, graphene scaffolds and films have also been used to culture mesenchymal cells, and isolated exosomes from them can be used as a treatment in a preclinical model of AD (mutant *APP* mice) [144]. This provides a different application of 3D devices in the management of diseases, effectively rescuing Aβ pathology.

The use of 3D scaffolds still relies on cells cultured in defined media, which cannot always be precisely controlled, especially if variables like precise medium composition (gradient generation), changing substrates, or spatial cell arrangements are to be tested (Figure 4b). Hence, microfluidic devices have been developed that improve reproducibility and allow the possibility of automatising and upscaling experimental work. Also, 3D cultures in a microfluid platform have been shown to provide an interesting strategy platform to allow the use of different cell types (microglia, astrocytes, and neurons) (reviewed in [145]). These have been shown to improve DA differentiation, reducing the timing to generate mature DA neurons and the efficiency in generating them [146,147]. For PD, these devices have been employed to study α-Syn accumulation [148] and antibody-based therapies [149]. For AD, microfluidic devices have been used to study neuronal death in an inflammatory context [150]. More complex microfluidic 3D devices have been used in PD genetic models to generate the so-called organs-on-chip [82,146,151]. Efforts are being made to culture astrocytes in microfluidic devices to study their role in PD using an organ-on-chip model [152]. Another group developed an organ-on-chip representative of DA neurons to include astrocytes and microglia, among other cells [153].

Another engineering-based approach to model 3D cell culture is 3D bioprinting (Figure 4c). Differently to scaffolds, in 3D bioprinting, cells and scaffolds are deposited in a single step. Furthermore, it allows a precise cell arrangement, and even multicellular compositions to better mimic the in vivo tissue. For PD, this approach allows the different cell populations involved in the disease to be co-cultured [154]. The authors deposit a hydrogel-based 3D system together with neural progenitor cells that are induced to differentiate into DA neurons. This bioprinted 3D structure is then co-cultured with astrocytes and monocyte-derived macrophages in transwells to better model PD as it includes cell interactions between all cell types involved in the disease. Other authors combined 3D bioprinting with scaffolding to generate a mouse whole-brain model composed of midbrain neurons, including DA neurons [43]. In this 3D system, PD is induced by 6-OHDA treatment. This methodology has also been used to model AD using iPSCs from patients [155].

Advantages and Disadvantages of Engineering-Based 3D Models:

These innovative 3D models are generated from an engineering point of view. Hence, their focus is reproducibility, aiming to facilitate high-throughput production and readouts (reviewed in [156]). Through these approaches, researchers can control the 3D model structure and nutrient supply to achieve homogeneous cell behaviour. However, in PD and AD research, we are quite far away from accomplishing this.

Currently, bioengineering approaches are limited to those biology labs with interdisciplinary links to engineers producing the scaffolds, microfluids, and bioprinting devices. Likewise, engineers interested in generating devices to study AD or PD need to develop links to biology labs or incorporate biologists in their teams capable of culturing and differentiating the required cell types. This can be a handicap. Furthermore, both teams need to communicate and develop a common language that both understand to progress. Given the importance of this approach and the need for standardised scaffolding, several companies are focusing their efforts on developing hydrogels with properties comparable to Matrigel but with less batch-to-batch variability, like hydrogels, based on synthetic peptides.

At present, the main problem with most published studies using bioengineering approaches is that they mainly only focus on culturing one cell type (neural). Others have developed scaffold-based 3D cultures of astrocytes or microglia (reviewed in [157]). Ideally, and to better model PD, one should use multiscaffold-based 3D cultures of all of these cell types together with DA neurons and biosensors to generate a fully functional organ with astrocytes together with cortical neurons [158].

## 5. Challenges and Future Perspectives

No current system to model PD or AD in vitro, either 2D or 3D, is perfect, but clear advances are being made in designing and developing new systems to better represent the physiopathological cellular characteristics of both diseases (Table 1). The ideal model should include all known cell types involved in PD or AD together with their architecture and interactions. This is not easy to accomplish, as each cell type has specific and distinct culture conditions. As mentioned above, recent advances in tissue engineering and biomaterial fabrication technologies have allowed the development of more sophisticated and physiologically relevant models. However, the assembly of multidisciplinary and interdisciplinary teams, composed of researchers with experience in multiple cell lines along with engineers with an interest in cellular processes, is challenging. Furthermore, these approaches require substantial funding, which is not always at hand in all research laboratories. Therefore, simpler in vitro models may be more inaccurate, but they remain valid tools to investigate different distinct aspects of PD and AD, especially for high-throughput approaches. Although new 3D systems are already being developed that will allow the study of more complex biological processes, further work is still needed. We foresee that innovative studies using these novel preclinical tools will be soon published to elucidate new pathological mechanisms underlying PD and AD. However, without doubt, any research undertaken in in vitro models should be validated in an in vivo model, especially regarding behavioural alterations associated with these diseases.

## Figures and Tables

**Table 1:** Two- and three-dimensional model summary.

Dimensions	Name	Species	Biological Origin	Advantages	Disadvantages
2D	SH-SY5Y cells	Human	Neuroblastoma	Ease to differentiate into neurons Cost-effectiveness Batch-to-batch consistency	May not accurately replicate neurons
	PC12 cells	Rat	Pheochromocytoma tumour	Ease to differentiate into neurons Cost-effectiveness Batch-to-batch consistency	May not accurately replicate neurons Rat origin
	LUHMES cells	Human	Foetal mesencephalon	Ease to differentiate into neurons Cost-effectiveness Batch-to-batch consistency	May not accurately replicate neurons
	iPSCs-derived cells	Human	(Usually) fibroblasts	More accurate representation of neuronal physiology	Variability in differentiation protocol efficiency High costs Technical complexity Cell-to-cell line variability
	Induced neurons	Human	(Usually) fibroblasts	More accurate representation of neuronal physiology Faster differentiation to neurons than iPSCs	Variability in differentiation protocol efficiency High costs Technical complexity Cell-to-cell line variability Poor long-term proliferation
3D	Organoids	Human	(Usually) iPSCs	Better replicate the in vivo architecture Multiple cell types present	Poor long-term culture High variability (poor reproducibility) Poor ageing modelling capability
	Engineering-based 3D models	Multiple (usually human)	Multiple cell types can be used	More physiologically relevant connections High reproducibility Long-term culture	Interdisciplinary links required

## Data Availability

No new data were created or analyzed in this study. Data sharing is not applicable to this article.

## Abbreviations

Aβ	amyloid-β
ACh	cholinergic
AD	Alzheimer’s disease
APP	amyloid precursor protein
DA	dopaminergic
GSK-3β	glycogen synthase kinase-3β
iNs	induced neurons
iPSCs	induced pluripotent stem cells
ipsNSCs	neural stem cells differentiated from iPSCs
LRRK2	leucine rich repeat kinase 2
LUHMES	Lund human mesencephalic
MO	midbrain organoid
MPTP	1-methyl-4-phenyl-1,2,3,6-tetrahydroperydine
NFT	neurofibrillary tangles
ND	neurodegenerative disease
PINK1	PTEN-induced kinase 1
PRKN	Parkin
PD	Parkinson’s disease
PP1	phosphatase 1
PSEN	presenilin
RA	retinoic acid
SNCA	synuclein alpha
VPS35	vacuolar sorting protein 35
6-OHDA	6-hydroxydopamine
